# Predictive impact of polymorphism of PNPLA3 on HCC development after interferon therapy in Japanese patients with chronic hepatitis C

**DOI:** 10.1186/2193-1801-2-251

**Published:** 2013-06-01

**Authors:** Yuki Moritou, Fusao Ikeda, Yoshiaki Iwasaki, Nobuyuki Baba, Kouichi Takaguchi, Tomonori Senoh, Takuya Nagano, Yasuto Takeuchi, Tetsuya Yasunaka, Hideki Ohnishi, Yasuhiro Miyake, Akinobu Takaki, Kazuhiro Nouso, Kazuhide Yamamoto

**Affiliations:** Department of Gastroenterology and Hepatology, Okayama University Graduate School of Medicine, Dentistry and Pharmaceutical Sciences, 2-5-1, Shikata-cho, Okayama, 700-8558 Japan; Department of Molecular Hepatology, Okayama University Graduate School of Medicine, Dentistry and Pharmaceutical Sciences, Okayama, Japan; Health and Environment Center, Okayama University, Okayama, Japan; Department of Internal Medicine, Kagawa Prefectural Central Hospital, Takamatsu, Japan

**Keywords:** PNPLA3, Interferon, HCC, HCV

## Abstract

The impact of single-nucleotide polymorphisms (SNP) of patatin-like phospholipase domain-containing protein 3 (PNPLA3) on development of hepatocellular carcinoma (HCC) is not clarified for Japanese patients with chronic hepatitis C. The present study investigated the associations of rs738409 PNPLA3 with HCC development after the antiviral therapy with peg-interferon and ribavirin for Japanese patients with hepatitis C virus serotype 1 and high viral load. Of the 271 patients enrolled in the study, 20 patients developed HCC, during a median follow-up period of 4.6 years. Multivariate analysis in the proportional hazards models revealed that sex, body mass index, platelet counts, and alpha feroprotein (AFP) had significant associations with HCC development (*p* = 0.011, 0.029, 0.0002, and 0.046, respectively). Multivariate regression analysis revealed that PNPLA3 148 M was significantly associated with serum AFP level (*p* = 0.032), other than body mass index, platelet count, and alanine aminotransferase (*p* = 0.0006, 0.0002, and 0.037, respectively), and that serum AFP level was significantly associated with PNPLA3 148 M (*p* = 0.017). Serum AFP level is an important factor in predicting HCC development after the antiviral therapy for Japanese patients with chronic hepatitis C, the mechanism of which might involve its significant associations with the SNP genotype of PNPLA3.

## Introduction

Hepatitis C virus (HCV) infection causes chronic hepatitis, and may progress to liver cirrhosis and hepatocellular carcinoma (HCC). More than 170 million people worldwide are infected with HCV, creating a serious global health problem (Kato 2001). Combination therapy with pegylated interferon alpha and ribavirin has a sustained virological response (SVR) in 50% of patients with HCV genotype 1 (Firpi & Nelson 2007). Recent therapeutic regimens using direct-acting antiviral agents have improved the SVR up to 80% (Ghany et al. 2011a; Zeuzem et al. 2011; Kumada et al. 2012).

Recently, the single-nucleotide polymorphism (SNP) of rs738409 patatin-like phospholipase domain-containing protein 3 (PNPLA3) has been identified for its significant associations with liver steatosis and fibrosis in patients with fatty liver disease and alcoholic liver disease (Romeo et al. 2008; Valenti et al. 2010). As for patients with chronic hepatitis C, associations of this SNP genotype with liver steatosis, fibrosis, and the outcome of interferon therapy have also been suggested (Trépo et al. 2011; Valenti et al. 2011). These results were mostly obtained from the analyses of patients of European origins; however, analysis of Japanese patients could yield somehow different results. A recent genome-wide association study for Japanese patients with non-alcoholic fatty liver disease by Kawaguchi et al. revealed that the risk variant of PNPLA3 148 M is significantly associated with non-alcoholic steatohepatitis, but did not show significant differences in liver steatosis or fibrosis among the patients with non-alcoholic fatty liver disease (Kawaguchi et al. 2012). Miyashita et al. studied the associations of the SNP genotype of PNPLA3 with liver fibrosis and inflammation for Japanese patients with chronic hepatitis C, without significant associations in the results (Miyashita et al. 2012). Takeuchi et al. reported that the SNP genotype of PNPLA3 might not affect HCC prognosis in Japanese patients with hepatitis B virus, HCV, or non-alcoholic fatty liver diseases (Takeuchi et al. 2013). Thus, it is possible that ethnic differences exist in the genetic background of the pathogenesis of chronic liver diseases.

Prediction or early detection of HCC development after antiviral therapy is increasingly needed because of recent improvement of antiviral therapy. The present study investigated the associations of the SNP genotype of PNPLA3 with HCC development after interferon therapy in Japanese patients with chronic hepatitis C, in order to clarify the predictive impact of the SNP genotype of PNPLA3 on HCC development in Japanese patients with chronic hepatitis C.

## Methods

### Patients

The present study enrolled 271 Japanese patients suffering from chronic hepatitis C with HCV serotype 1 and a high viral load 5.0 log IU/mL in real-time PCR. All patients received 48 weeks of antiviral therapy with standard doses of pegylated interferon alpha-2a (180 μg/week) or 2b (1.5 μg/body weight/week) with ribavirin (600-1000 mg/day) at either Okayama University Hospital or Kagawa Prefectural Central Hospital. The outcomes of the interferon therapy were evaluated, according to the practice guidelines by the American association for the study of liver diseases, and the Japan society of hepatology (Ghany et al. 2011b). Hepatocellular carcinoma was ruled out using dynamic computed tomography, magnetic resonance imaging or ultrasonography prior to the start of the interferon therapy. Heavy alcohol-drinking behavior was defined as daily alcohol intake >70 grams. Patients with hepatitis B virus co-infection, human immunodeficiency virus co-infection, or autoimmune liver disease were not included in the study. The study was performed in accordance with the Helsinki Declaration, and the protocols were approved by the ethics committees of the participating institutes. All patients provided informed consent before enrolment into the study.

### Diagnosis of liver histology

Liver histology was evaluated for 219 patients prior to the start of the interferon therapy. Liver fibrosis stage and hepatitis activity grade were assigned for all patients by two pathologists according to the criteria of Desmet et al. ( 1994). The severity grade of hepatic steatosis was defined according to the criteria used by Valenti et al. ( 2011).

### Genotyping of single nucleotide polymorphism

Genomic DNA was extracted from whole-blood samples by means of a QIAamp DNA Mini Kit according to the manufacturer’s protocol (Qiagen, Tokyo, Japan). The SNPs of rs8099917 IL28B, and rs738409 PNPLA3 were genotyped using the TaqMan predesigned SNP genotyping assays, as recommended by the manufacturer (Applied Biosystems, Tokyo, Japan). The SNP genotypes of all the samples could be obtained with these systems.

### Variations of the amino acid 70 of HCV core

For the 241 patients in the study, variations of the amino acid 70 of HCV core (arginine or glutamine) were determined with direct sequence method. The amplifications of HCV core in the serum were performed as previously reported (Ikeda et al. 2010), and direct sequencing was carried out by Big Dye termination cycle sequencing using an ABI Prism 310 genetic analyzer (Applied Biosystems, Foster City, CA).

### Follow-up and HCC diagnosis

In accordance with the clinical practice manual from the Japan Society of Hepatology (Makuuchi et al. 2010), HCC diagnosis was done using ultrasonography, computed tomography, magnetic resonance imaging, hepatic angiography, and/or tumor biopsy, in combination with the detection of serum levels of alpha-fetoprotein (AFP), AFP-L3, and des gamma-carboxy prothrombin (DCP). Only the patients without HCC at 1 year or longer after interferon therapy were included in the study. This follow-up period was defined as the time between the cessation of interferon therapy and HCC diagnosis or the latest confirmation of survival.

### Statistical analysis

Data are expressed as means ± standard deviations. Proportional hazards models were utilized to identify the factors associated with HCC development during the follow-up period after interferon therapy. The incidences of HCC development were also estimated using the Kaplan–Meier method and compared with the log-rank test. Factors associated with the SNP genotype of PNPLA3 and AFP were analyzed by stepwise logistic regression analysis. A value of *p* <0.05 was considered significant. Statistical analysis was performed with JMP software (SAS Institute, Cary, NC).

## Results

### Patient characteristics of the patients enrolled in the study

**Figure 1:**
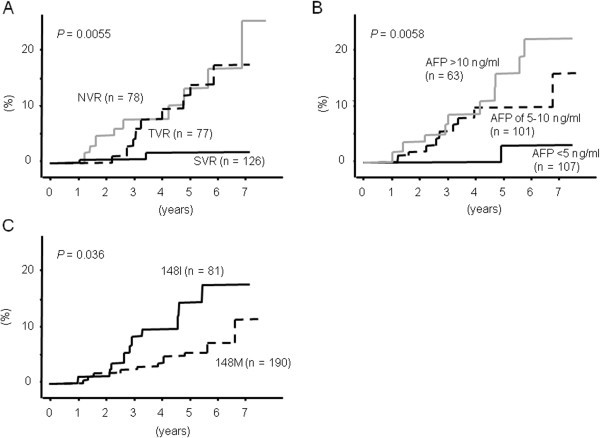
**The incidence of HCC development after interferon therapy.** The incidence of HCC development after interferon therapy was presumed by the Kaplan–Meier method for each group by dividing the patients by the outcomes of the interferon therapy (**A**), serum levels of alpha fetoproteins prior to the interferon therapy (**B**), and the SNP genotypes of PNPLA3 (**C**).

The characteristics of patients enrolled in the study are shown in Table 1. Their average age was 57 years, and 131 patients (48%) were female. Interferon therapy resulted in sustained virological responses in 126 patients (46.5%), relapse in 77 patients, and null or partial virological response (NVR) in 68 patients. During the follow-up after the interferon therapy (median period of 4.6 year), the development of HCC was observed in 20 patients (16%). The results of AFP-L3 prior to interferon therapy were undetectable or below the lower limit of quantification for all patients. Consistent with previous reports (Makuuchi et al. 2010; He et al. 2009; Nishiguchi et al. 2001), SVR contributed to lowering the risk of HCC development (Hazards ratio 0.13, *p* = 0.0067, Table 2), while patients with NVR showed a high incidence of HCC (Hazards ratio 2.4, *p* = 0.048). The incidences of HCC development were compared among patient groups formed according to interferon therapy outcomes (Figure 1A). The NVR patients had much higher incidence of HCC development than the SVR or relapse patients (*p* = 0.0055, the log-rank test). The SVR and relapse patients had similar risks of HCC development during the first 2 years after interferon therapy, while relapse patients had increased risk thereafter.

**Table 1:** **Characteristics of the patients enrolled in the study**

Patient characteristics	N = 271
Age (years)	57 ± 10^†^
Sex (male/female)	140/131
Body mass index	24 ± 3^†^
Liver fibrosis (Stage 1/2/3/4)	71/82/60/6
Hepatitis activity (Grade 1/2/3)	164/49/6
rs 8099917 of IL28B (TT/TG/GG)	198/72/1
rs 738409 of PNPLA3 (CC/CG/GG)	81/136/54
HCV core amino acids 70 (arginine/glutamine)	150/91
Alanine aminotransferase (IU/L)	66 ± 55^†^
Platelet count (10000 /mm^3^)	17 ± 6^†^
Alpha fetoprotein (ng/mL)	11 ± 19^†^
HOMA-IR	4.2 ± 8.4^†^
Therapeutic outcome (SVR/relapse/NVR)	126/77/68

^†^: Mean ± standard deviation; PNPLA3, patatin-like phospholipase domain-containing protein 3; HOMA-IR, homeostasis model assessment insulin resistance; SVR, sustained virological response; NVR, null or partial virological response.

**Table 2:** **Proportional hazards model for the development of hepatocellular carcinoma**

	Univariate analysis		Multivariate analysis	
Factors	Hazards ratio (range^†^)	***p***	Hazards ratio (range^†^)	***p***
Age (years)	1.0 (0.99-1.1)	0.12		
Sex (1: male)	5.6 (1.6-19)	0.0062	5.5 (1.5-21)	0.011
Body mass index	1.2 (1.0-1.3)	0.0086	1.2 (1.0-1.3)	0.029
Liver fibrosis	3.8 (1.9-7.3)	<0.0001		
Hepatitis activity	1.1 (0.44-2.6)	0.88		
Liver steatosis (>30%)	2.1 (0.95-4.8)	0.067		
rs 8099917 of IL28B (TT)	0.62 (0.25-1.6)	0.31		
rs 738409 of PNPLA3 (148 M)	0.40 (0.17-0.97)	0.043		
Amino acid 70 of HCV core (glutamine)	3.4 (0.86-14)	0.082		
Alanine aminotransferase (>40 IU/L)	1.2 (0.68-2.2)	0.51		
Platelet count (10^4^/mm^3^)	0.77 (0.69-0.87)	<0.0001	0.76 (0.66-0.88)	0.0002
Alpha fetoprotein (ng/mL)	1.1 (1.0-1.1)	0.0002	1.1 (1.0-1.1)	0.046
HOMA-IR	0.97 (0.79-1.2)	0.76		
Sustained virological response	0.13 (0.03-0.57)	0.0067		
Null or partial virological response	2.4 (1.0-5.9)	0.048		

^†^: 95% confidence interval; PNPLA3, patatin-like phospholipase domain-containing protein 3; HOMA-IR, homeostasis model assessment insulin resistance.

### Patient characteristics associated with HCC development after interferon therapy

We compared the patient characteristics related to HCC development in proportional hazards models (Table 2). Male gender, high body mass index (BMI), advanced liver fibrosis, low platelet counts, and high levels of AFP had significant associations with HCC development in univariate analysis (Hazards ratio 5.6, *p* = 0.0062, Hazards ratio 1.2, *p* = 0.0086, Hazards ratio 3.8, *p* <0.0001, Hazards ratio 0.77, *p* <0.0001, and Hazards ratio 1.1, *p* = 0.0002, respectively). The incidence of HCC development was significantly lower in the patients with PNPLA3 148 M than in those with PNPLA3 148I (Hazards ratio 0.4, *p* = 0.043). Liver steatosis and amino acid 70 of HCV core affected HCC development slightly (Hazards ratio 2.1, *p* = 0.067, and Hazards ratio 3.4, *p* = 0.082, respectively), while the SNP genotype of IL28B was not associated with the risk of HCC development (Hazards ratio 0.62, *p* = 0.31). Stepwise multivariate analysis revealed that male gender, high BMI, low platelet counts, and high levels of AFP had significant associations with HCC development (Hazards ratio 5.5, *p* = 0.011, Hazards ratio 1.2, *p* = 0.029, Hazards ratio 0.76, *p* = 0.0002, and Hazards ratio 1.1, *p* = 0.046, respectively).

### PNPLA3 148 M is independently associated with low levels of AFP

Serum AFP levels prior to the interferon therapy were significantly associated with HCC development after interferon therapy (*p* = 0.0058, the log-rank test, Figure 1B). Especially, patients with AFP <5 ng/mL prior to interferon therapy showed significantly lower incidence of HCC development than those with AFP ≥5 ng/mL (Hazards ratio 0.09, *p* = 0.021). We further examined the patient characteristics prior to the interferon therapy regarding associations with serum AFP by stepwise logistic regression analysis (Table 3). The results showed several significant factors associated with serum AFP, such as BMI, platelet count, and alanine aminotransferase (*p* = 0.0006, 0.0002, and 0.037, respectively). The SNP genotype of PNPLA3 was also an independent factor associated with serum AFP (*p* = 0.032). Therefore, we studied the patient characteristics associated with the PNPLA3 148 M genotype (Table 4). Low levels of AFP >5 ng/mL were significantly associated with PNPLA3 148 M in stepwise multivariate logistic regression analysis (*p* = 0.017). Advanced liver fibrosis was also associated with PNPLA3 148 M in univariate analysis (*p* = 0.028), although this association did not show statistical significance in multivariate analysis (*p* = 0.20).

**Table 3:** **Stepwise logistic regression analysis of the factors related to alpha fetoprotein > 5 ng/mL**

	Univariate analysis		Multivariate analysis	
Factors	Odds ratio (range^†^)	***p***	Odds ratio (range^†^)	***p***
Age (years)	1.0 (1.0-1.1)	0.0024	1.0 (1.0-1.1)	0.064
Sex (1:male)	0.95 (0.58-1.6)	0.84		
Body mass index	1.2 (1.1-1.3)	0.0005	1.2 (1.1-1.3)	0.0006
Liver fibrosis	2.8 (1.9-4.2)	<0.0001		
Hepatitis activity	1.1 (0.61-1.9)	0.83		
Liver steatosis (1: >30%)	1.8 (1.1-3.0)	0.026	1.3 (0.72-2.4)	0.37
rs 8099917 of IL28B (1: TT)	1.1 (0.65-2.0)	0.67		
rs738409 of PNPLA3 (1: 148 M)	0.36 (0.20-0.65)	0.0007	0.46 (0.22-0.93)	0.032
Amino acid 70 of HCV core (1: glutamine)	1.1 (0.58-2.2)	0.71		
Alanine aminotransferase (1: >40 IU/L)	2.3 (1.6-3.3)	<0.0001	1.6 (1.0-2.4)	0.037
Platelet count (1: >1.5x10^5^/mm^3^)	0.33 (0.22-0.50)	<0.0001	0.34 (0.19-0.60)	0.0002
HOMA-IR	1.0 (0.97-1.1)	0.33		

^†^: 95% confidence interval; PNPLA3, patatin-like phospholipase domain-containing protein 3; HOMA-IR, homeostasis model assessment insulin resistance.

**Table 4:** **Stepwise logistic regression analysis of the factors related to PNPLA3 148 M**

	Univariate analysis		Multivariate analysis	
Factors	Odds ratio (range^†^)	***p***	Odds ratio (range^†^)	***p***
Age (years)	0.98 (0.95-1.0)	0.12		
Sex (1: male)	0.69 (0.41-1.2)	0.17		
Body mass index	1.0 (0.93-1.1)	0.73		
Liver fibrosis	0.68 (0.48-0.96)	0.028	0.78 (0.54-1.1)	0.20
Hepatitis activity	1.3 (0.70-2.3)	0.43		
Liver steatosis (1: >30%)	0.96 (0.55-1.7)	0.87		
Amino acid 70 of HCV core (1: glutamine)	0.80 (0.38-1.7)	0.56		
Alanine aminotransferase (1: >40 IU/L)	0.72 (0.51-1.0)	0.068		
Platelet count (1: >1.5x10^5^/mm^3^)	2.1 (0.93-4.7)	0.076		
Alpha fetoprotein (1: >5 ng/mL)	0.36 (0.20-0.65)	0.0007	0.42 (0.21-0.85)	0.017
HOMA-IR	0.18 (0.92-1.0)	0.18		

^†^: 95% confidence interval; PNPLA3, patatin-like phospholipase domain-containing protein 3; HOMA-IR, homeostasis model assessment insulin resistance.

## Discussion

The impact of the SNP of PNPLA3 on interferon therapy and HCC development after interferon therapy is still undetermined for Japanese patients with chronic hepatitis C. The present study investigated the factors associated with HCC development after interferon therapy for Japanese patients with chronic hepatitis C, and is the first to clarify significant associations of the SNP of PNPLA3 with HCC development after interferon therapy.

The incidence of HCC development was significantly lower in the patients with PNPLA3 148 M than in those with PNPLA3 148I (Table 2, and Figure 1C). Considering the roles of the SNP genotype of PNPLA3 with HCV-related HCC, a previous report by He et al. revealed impaired triglyceride hydrolysis in those with PNPLA3 148 M (He et al. 2009), which might work against direct regulation of HCV in hepatic lipid metabolism, and suppress HCC development. We explored clinical characteristics as they related to the SNP genotype of PNPLA3. In general, advanced liver fibrosis, high age, and male gender have been reported as risk factors of HCC development for patients with chronic hepatitis C (Nishiguchi et al. 2001; Imai et al. 1998; Yoshida et al. 1999). Liver steatosis, inflammation, insulin resistance, and excessive body composition may accelerate HCC development. Among those factors, we could find none significantly associated with the SNP genotype of PNPLA3 as previously reported (Miyashita et al. 2012; Kotronen et al. 2009).

Serum AFP was only the factor we found to be significantly associated with the PNPLA3 genotype in the present study; patients with PNPLA3 148 M had lower levels of serum AFP than those with PNPLA3 148I. Meanwhile, multiple factors such as advanced liver fibrosis, inflammation, and body composition were significantly associated with serum AFP. Only the SNP genotype of PNPLA3 is the significant factor that is genetically determined. The associations of serum AFP with HCC development and the mechanisms by which the SNP genotypes of PNPLA3 contribute to serum AFP are still unknown, and need to be clarified in a future study.

Recent studies on Caucasian patients have shown that PNPLA3 148 M was significantly associated with liver steatosis (Poynard et al. 2003; Valenti et al. 2012; Clark et al. 2012), while the present study on Japanese patients showed no significant impact of the SNP genotype of PNPLA3 on liver steatosis for the patients with chronic hepatitis C. The high distribution of PNPLA3 148 M in Japanese patients might be associated with ethnic differences in the role of PNPLA3 in the pathogenesis of chronic liver disease; in the Hap Map study the prevalence of PNPLA3 148 M was found to be 43% in the Japanese, much higher than its prevalence in Caucasians (23%).

In conclusion, serum AFP level is an important factor in predicting HCC development after the antiviral therapy for Japanese patients with chronic hepatitis C, the mechanism of which might involve its significant associations with the SNP genotype of PNPLA3.

## Abbreviations

HCV: Hepatitis C virus
HCC: Hepatocellular carcinoma
SVR: Sustained virological response
SNP: Single-nucleotide polymorphisms
PNPLA3: Patatin-like phospholipase domain-containing protein 3
AFP: Alpha fetoprotein
DCP: Des gamma-carboxy prothrombin
NVR: Null or partial virological response
BMI: Body mass index.
